# Impact of altering proximity on snack food intake in individuals with high and low executive function: study protocol

**DOI:** 10.1186/s12889-016-3184-9

**Published:** 2016-06-13

**Authors:** Jennifer A. Hunter, Gareth J. Hollands, Dominique-Laurent Couturier, Theresa M. Marteau

**Affiliations:** Behaviour and Health Research Unit, University of Cambridge, Institute of Public Health, Forvie Site, Robinson Way, Cambridge, CB2 0SR UK

**Keywords:** Proximity, Placement, Executive function, Socio-economic position, Snack food, Dietary behaviour

## Abstract

**Background:**

Despite attempts to improve diet at population level, people living in material and social deprivation continue to consume unhealthy diets. Executive function - the ability to regulate behaviour and resist impulses – is weaker in individuals living in deprivation. Dietary interventions that educate and persuade people to reflect on and actively change behaviour may therefore disproportionately benefit individuals who are socioeconomically advantaged and have stronger executive function, thus exacerbating inequalities in health resulting from unhealthy diets. In contrast, manipulating environmental cues, such as how far away a food is placed, does not appeal to reasoned action and is thought to operate largely outside of awareness to influence behaviour. People eat more of a food when it is placed closer to them, an effect seemingly robust to context, food quality and body-weight status. However, previous studies of this ‘proximity effect’ are limited by small samples consisting mainly of university staff or students, biased towards higher socio-economic position and therefore likely stronger executive function. This study aims to test the hypothesis that placing food further away from a person decreases intake of that food regardless of executive function.

**Methods/Design:**

156 members of the general public, recruited from low and high socio-economic groups, will be randomised to one of two conditions varying in the proximity of a snack food relative to their position: 20 cm or 70 cm. Participants are told they will be taking part in a relaxation study – and are fully debriefed at the conclusion of the session. The primary outcome is the proportion of participants eating any amount of snack food and the secondary outcome is the mean amount eaten. Executive function is assessed using the Stroop task.

**Discussion:**

The proposed study takes a novel step by investigating the effect of proximity on snack food intake in a general population sample consisting of those with high and low executive function, appropriately powered to detect the predicted proximity effect. If this effect occurs irrespective of executive function and socio-economic position, it may have potential to reduce inequalities patterned by socio-economic position if implemented in real-world settings such as shops or restaurants.

**Trial registration:**

Registered with the ISRCTN registry: ISRCTN46995850 on 07 October 2015.

**Electronic supplementary material:**

The online version of this article (doi:10.1186/s12889-016-3184-9) contains supplementary material, which is available to authorized users.

## Background

The global burden of disease, including diabetes mellitus and various cancers, can be attributed in part to unhealthy diet [1]. Furthermore, non-cardio-vascular disease and non-cancer mortality rates tend to be higher in populations characterised as of low socio-economic position (SEP) [2], which can be explained largely by unhealthy behaviours, including consumption of a less healthy diet compared to high SEP groups [2]. However, interventions to address this problem, which involve educating recipients about their health behaviours, are less likely to benefit disadvantaged populations [3–5]. Other interventions are therefore needed to effectively improve dietary behaviour in low SEP groups. The objective of the proposed study is to test the hypothesis that placing food further away from a person decreases intake of that food – the ‘proximity effect’ - and assess whether this effect is evident in a general population sample regardless of individual differences in EF. It is hypothesised that manipulation of environmental cues will be effective regardless of EF; the implications of which are discussed in relation to the development of population-level dietary interventions.

Choice architecture interventions to change health behaviour in micro-environments involve altering the properties and placement of objects or stimuli, such as food products [6]. One way in which the environment can be manipulated to change behaviour is through altering the proximity of food products; individuals consistently select and consume more of a food that is within easy reach compared to when it is placed further away [7–17]. This ‘proximity effect’ remains consistent across many environments, such as in cafeterias [10–12, 17], shops [13], offices [7, 9] and kitchens [14, 15] and occurs regardless of food characteristics such as calorie content [11, 14] – see Table 1 in Additional file 1 for details of these studies. Furthermore, this effect does not appear to differ by BMI, levels of craving, food preferences or body-weight [8, 14, 16].

Engineering aspects of the physical environment could be used to shape eating behaviour [18, 19]. The apparent robustness of the proximity effect could be used, for example, to develop dietary interventions by placing less healthy foods further away from people and thus making them less easy to select, or placing more healthy foods closer to increase the chances that they are selected. The level of effort required to obtain a product may act as an underlying mechanism, with the least effortful course the most likely, resulting in decreased intake of less healthy foods without the need for explicit instruction or conscious deliberation [20, 21]. Increasing the distance of a less healthy food by as little as 10 in. in a cafeteria servery is enough to reduce intake of the food, since this increase in distance requires greater effort to reach for the food [10]. In one study in which perceived effort was measured as a postulated mechanism underlying the proximity effect, participants perceived snacks placed further away as requiring more effort to obtain [8]. The proposed study will include measures of perceived effort to investigate this potential underlying mechanism. Perceived salience of snack-food has also been found to influence consumption of snacks, with participants who rate snacks as more tempting and noticeable consuming more of these snacks [8], which will also be assessed here.

In addition to testing the effect of food proximity on eating behaviour, the proposed study will also assess whether this effect is moderated by executive function.

### Executive function and socio-economic position

Executive function (EF) is an overarching term referring to a collection of top-down mental processes involved in behavioural control. These processes encompass inhibitory control of impulses, the flexibility to change mental states and updating memory of existing knowledge, each allowing us to monitor and control our behaviour [22–24]. The Reflective-Impulsive Model (RIM) postulates that behaviour is shaped via two distinct but interacting pathways [25]. The reflective pathway comprises actions towards identified goals resulting from reasoned, deliberative processes and reflecting an individual’s values. The impulsive system comprises actions resulting from the appraisal of external stimuli via non-conscious associative processes. EF resources determine the relative influence of these systems, with the reflective system thought to be predominant when there are sufficient motivational or self-regulatory resources available [26].

People differ significantly in EF strength, reflecting differences in genetics [27] and early years environments [28, 29]. EF is associated with BMI in childhood and adolescents and a range of eating behaviours [30, 31]. Greater EF strength is associated with selection of higher quality food [32] and lower fatty food consumption [33]. EF has also been found to be associated with SEP, with time spent in poverty in childhood impacting negatively on EF strength [28]. Investigation into the brain morphology behind this association reveals significant differences by parental education and family income in the structure of areas supporting EF [29]. Few studies, however, consider the role of EF in explaining variation in the outcome of interventions. One study finds a strong negative association between EF strength and the amount of food eaten when participants are facilitated to eat, but not when restricted from eating, demonstrating moderation by EF on contextual cues to eat [34]. However, this study only tests instructive cues which likely target reflective processes; therefore, this form of intervention is liable to interference from individual differences in EF. To date, the hypothesis that manipulation of environmental cues shapes eating behaviour in all recipients, irrespective of EF, remains untested [35].

Those in lower SEP groups are more likely to have unhealthy diets [2] and therefore would benefit disproportionately from effective interventions to initiate and maintain dietary change. However, the effectiveness of interventions to improve health behaviour differs by SEP [3–5]. There is evidence that certain types of interventions either do not impact upon or further generate inequalities between SEP groups [5]; specifically, interventions that require individuals to actively make choices are less likely to benefit disadvantaged groups [3–5]. Given the association between SEP and EF, the effectiveness of the aforementioned interventions may differ by SEP group due to a moderation effect of EF.

Conversely, interventions that involve altering environmental cues are thought to operate largely outside of awareness to shape behaviour and so are unlikely to activate reflective processes [20]. Therefore EF, which enables the reflective pathway through controlling the relative influence of the impulsive system (as termed by the RIM [25]) should not moderate the impact of these interventions. Interventions making structural changes to the environment may benefit low SEP groups [4] since they may by-pass individual differences in EF. Further research is required to determine whether the aforementioned interventions effectively change behaviour in low SEP populations.

### Gaps in the literature

Most intervention studies on the proximity effect have been conducted with highly educated samples of students or university staff. Even in studies involving participants who are more representative of the general population, these rarely explore the differential effects of an intervention by SEP [3] and few studies consider the role of EF in explaining variation in outcomes of interventions. Vohs & Heatherton [36] did investigate self-control, a facet of EF, in relation to proximity of snack foods; however, they did not take into account baseline individual differences in self-control and did not consider SEP as a factor.

The proposed study is, to our knowledge, the first to recruit a general population sample to investigate moderation of the proximity effect by EF and SEP. Considering the finding that years living in poverty impacts negatively on EF [28] and EF strength is associated with eating behaviour [32, 33], it is important that EF is considered as a potential moderator influencing intervention outcomes and eating behaviour, especially when an intervention is targeted to low SEP groups, to avoid intervention generated inequalities [5]. In addition, this study may provide preliminary evidence for the hypothesis that choice architecture interventions operate via non-conscious mechanisms.

### Aims

To test the primary hypothesis that placing food further from a person decreases the likelihood that they take any food, and to test the secondary hypothesis that this effect is not moderated by EF.

### Hypotheses

1. Consumption of a snack food is less likely when it is placed further from participants
2. The proximity effect is not moderated by executive function

## Methods/Design

### Study design

The study is an experiment with a between-subjects design. Participants are randomly allocated to one of two proximity conditions:

1. Proximal snack: bowl placed 20 cm from participant
2. Distal snack: bowl placed 70 cm from participant

### Randomisation

Study appointments are assigned to one of the two experimental conditions using a randomly generated number sequence. The external research agency recruiting participants is responsible for allocating participants to the appointments and is blind to the randomisation of participants to the conditions. Likewise, the research team is blind to, and have no control over, the process of allocating participants to the appointments.

### Study setting

**Fig. 1:**
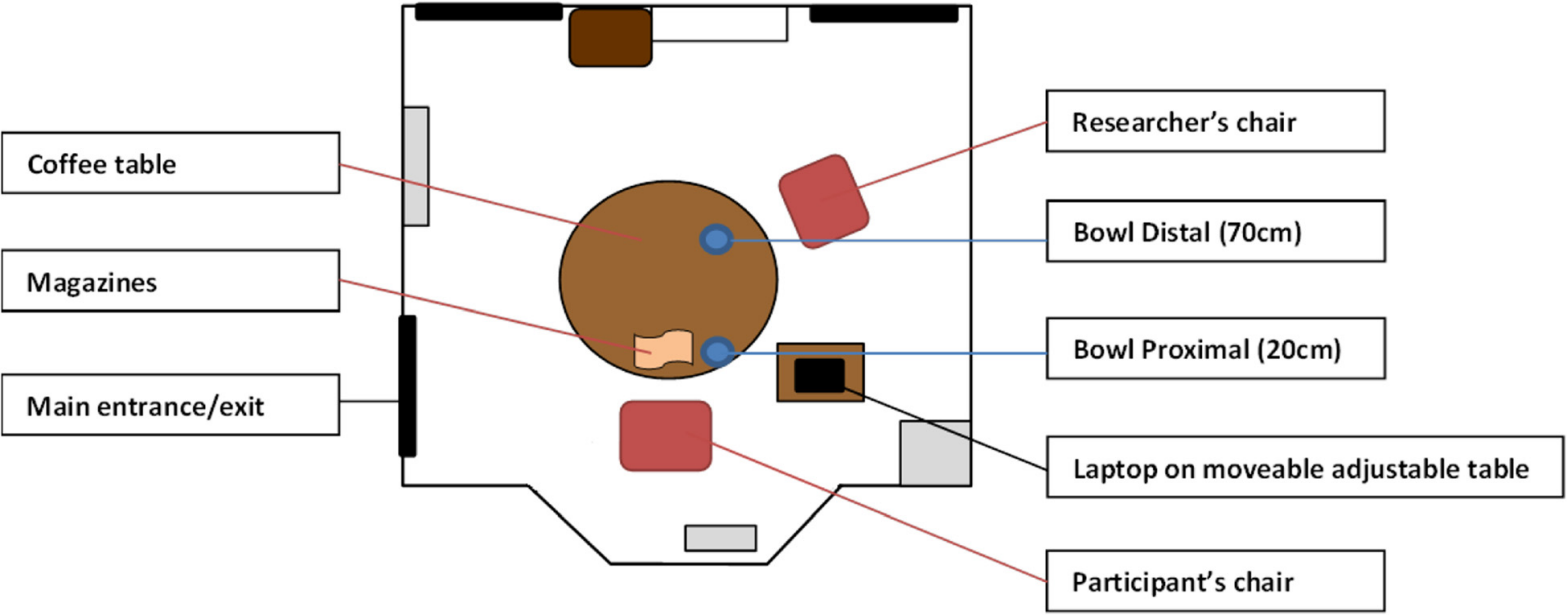
Map of testing room. The laptop is removed from the room during the relaxation period; the bowl and magazines are removed during testing periods while the laptop is being used

Participants are tested individually in an experimental session lasting approximately 45 min. The testing room measures 3.3 m by 3.9 m with a large table and chair; see Fig. 1 for layout of room.

### Participant recruitment

A research agency recruits participants from the general population via an online panel and the street (http://rootsresearch.co.uk/). Participants are eligible for inclusion if they are aged 18 years and over, and are excluded if they have any relevant food allergies or intolerance. Participants are reimbursed £25 for their time.

### Sample size calculation

Based on the aggregate results of two previous studies [8] giving probabilities of 0.76 in the proximal(near) condition and 0.39 in the distal(far) condition that participants will take the snacks, given a power of 80 % and a significance level of .05, we require 56 participants (28 in each study arm) to detect a main effect of proximity on our primary outcome (proportion of participants consuming any snacks) in a logistic regression, assuming a balanced sample. We intend, however, to collect data from a total of 156 participants, as this will increase the study power to detect an effect on the primary outcome. Though not powered on the secondary outcome, based on the effects observed in three similar studies [8, 14], this larger sample size may provide sufficient power to detect an effect on the secondary outcome (a continuous measure of mean intake of snacks). However, the latter is conditional on various assumptions being met, including the assumption that the distributions of the data are not overly affected by the inclusion of participants who consume nothing.

### Intervention

Distance of the snack bowl from the participant is manipulated: 1) Proximal = 20 cm 2) Distal = 70 cm. The increase in distance from 20 cm to 70 cm has been found to significantly affect intake of snack food; with this effect not increasing in strength with distances beyond 70 cm [8].

**Fig. 2:**
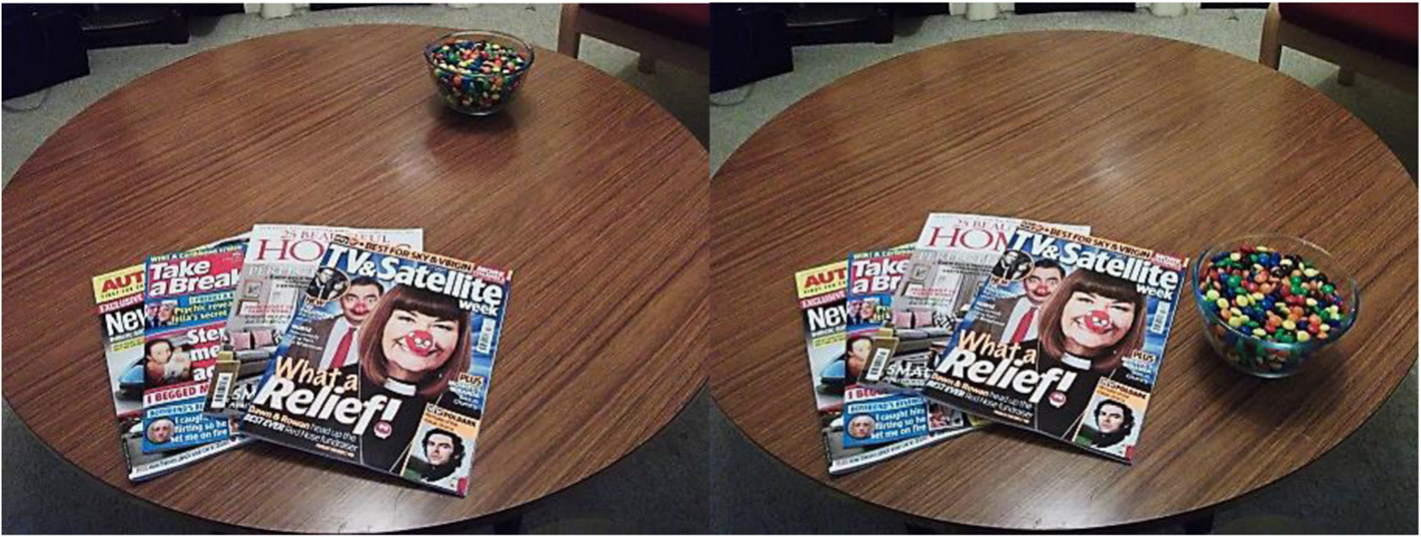
Snack food presentation. The left image shows the distal condition and the right image shows the proximal condition during the relaxation break. Images are taken from the participant’s perspective

1000 g of chocolate M&Ms (without peanuts) is used, the same portion size used in a previous study testing the proximity effect experimentally [8]. Chocolate M&Ms are chosen since chocolate receives high hedonic ratings across all groups [37]. The M&Ms are presented in an open one litre transparent bowl to ensure visibility of the snack. See Fig. 2 for snack food presentation.

### Measures

#### Primary behavioural outcome

##### Consumption of snack food as assessed by the proportion of participants consuming snacks (%)

Calculated from any difference in bowl weight from before to after the relaxation break.

#### Secondary behavioural outcome

##### Consumption of snack foods as assessed by the mean amount of snacks consumed (g)

Calculated from the difference in bowl weight (g) from before to after the relaxation break.

#### Executive function

The primary measure of EF is a state measure of response inhibition using the Stroop Test, administered using Inquisit 4 software. Self-report measures are used for additional analysis: see Table 2 in the Additional file 2 for details on items and order of presentation.

##### Response inhibition

Assessed by the Stroop Test [38] which has reliable and robust associations with dietary behaviour [39], correlates with other tests of inhibitory control [40] and is sensitive to detecting deficits in executive function and inhibitory control [32, 41]. The primary measure of response inhibition derived from the Stroop test is the interference score, calculated for each participant using latency data (incongruent trials – (congruent + control trials)/2).

This calculation for interference has been used widely as a primary outcome of the Stroop test since the trials first conducted by Stroop [38].

##### Self-reported EF

Assessed by WebEXEC, a 6-item self-report scale assessing the extent of problems individuals experience in every-day scenarios that involve executive functioning e.g. “Do you find it difficult to keep your attention on a particular task?” – see Table 2 in the Additional file 2 for all items. The items are rated using a four point scale labelled at either end: 1 = no problems experienced to 4 = a great many problems experienced. This scale achieves strong internal consistency, correlating strongly with the Dysexecutive Questionnaire (DEX) and behavioural tasks measuring EF [42]. A higher mean score indicates more problems and therefore poorer EF. Delay-discounting: Assessed by a binary single item measure requiring a choice between immediate receipt of £45 or receipt of £70 in three months [43]. Selection of the immediate smaller sum is indicative of weaker EF.

#### Other measures

##### Participant snack-bowl manipulation

Participants are not restricted from moving the bowl, which presents a potential issue whereby participants move the bowl and are then influenced by subsequent positions of the bowl rather than the initial position determined by the researcher as per protocol. This may affect the fidelity of the study. Any bowl distance manipulation by the participant is therefore recorded to enable assessment of the impact of moving the bowl on the effect of the proximity intervention.

##### Socio-economic position

Assessed by education level (highest qualification) as a dichotomous variable, with participants obtaining up to 5 or more GCSEs/1 A-level classified as low SEP and those obtaining bachelor degree/diploma and above classified as high SEP.

##### Ratings of general liking for chocolate

Assessed from ratings on a 100 unit visual analogue scale (VAS) to the statement “How pleasant would it be to experience a mouthful of chocolate now?”, anchored by “not at all” and “extremely” (adapted from [44]).

##### Hunger rating

Assessed using a 7-point rating scale anchored by 1 = “not at all” and 7 = “very” [45].

##### Perceived effort of accessing the snack food in the study

Assessed using a five-item measure of perceived effort to access the snack food, rated on a 5-point rating scale anchored by 1 = “completely disagree” and 5 = “completely agree” [8].

##### Perceived salience of the snack food used in the study

Assessed using a four-item measure of perceived salience of the snack food, completed on 5-point rating scales anchored by 1 = “completely disagree” and 5 = “completely agree” [8].

##### Handedness

Assessed using the four-item version of the Edinburgh Handedness Inventory [46] which assesses handedness for writing, throwing, using a toothbrush and using a spoon on a scale from 1 = always right to 5 = always left. This is measured to analyse whether snack-food intake is affected by handedness since the snack bowl is placed on the participants’ right side.

#### Awareness of intervention

As a cover story, participants are recruited on the basis that they are taking part in a study on relaxation and personality, in which snack food could be presented without suspicion [8]. Awareness of the proximity manipulation is assessed by responses to three questions: “What do you think the study was about?” What do you think the aim of the research is?” and “Did anything you were asked to do or anything that was in the room affect your actions or how you were thinking?”

### Procedure

The research agency recruits equal numbers of both low and high SEP participants based on education level in order to maximise chances of recruiting participants with high and low EF. On arrival to the session, participants are given the chance to read through the information sheet and complete a hard-copy of the consent form. Participants are asked to complete the Stroop, WebEXEC and delay-discounting measures on a laptop before being instructed to take a break for 10 min to relax.

A bowl of M&Ms is placed on the table either at 20 cm or 70 cm from the right arm of the participant (depending on randomisation). A selection of four magazines (varying in topic but none relating to food or health) is placed at the same time as the bowl, in a constant position on the table in front of the participant. The following instruction is given: “Since this is a study on relaxation and personality measures, we would like you to take a break for 10 min. Feel free to look through the magazines and help yourself to the M&Ms; I will be back in 10 min”. The researcher removes the laptop and leaves the room for 10 min. Upon re-entering the room the researcher removes the bowl of M&Ms and magazines. The bowl is then weighed and stored out of sight of the participant. Participants are asked to repeat EF measures to give credence to the cover story before completing the questionnaires. Questions pertaining to snack food are administered after participants are asked about their awareness of the intervention to prevent suspicion of the nature of the study earlier in the procedure. Participants are then debriefed and reimbursed for their time. See Table 2 in the Additional file 2 for order of questionnaire items.

### Analysis

Analyses conducted to test the two study hypotheses are as follows:

- Hypothesis 1. Consumption of a snack food is more likely when it is placed nearer to participants
  A Hurdle model is a two-part model in which the first stage models probability of participants taking any snacks (yes/no = binary) and the second stage models the amount of snacks eaten for participants who took any snacks (measured in grams = continuous). For the first stage, a logistic regression is conducted to investigate any between-group differences by condition in the primary outcome - the proportion of participants eating any snacks (%). In the second stage of the model, a General Linear Model (GLM) is used to assess between-group differences by condition in the secondary outcome – the mean intake of snacks (g). Effects of the intervention on the secondary outcome are examined for both the sub-sample of participants who take any amount of snacks (i.e. excluding those who do not take any snacks) and additionally for the full sample (thus including those who do not take any snacks).
- Hypothesis 2. The proximity effect is not moderated by executive function
  Interference score is entered into the analysis as the primary measure of EF. A logistic regression is conducted with proportion of participants taking snacks as the outcome and proximity as the predictor. An interaction term for proximity and EF is included in the analysis to investigate whether EF moderates the proximity effect.

A similar procedure is used to examine WebEXEC and delay discounting measures as additional indices of EF.

### Additional planned analysis

#### Moderation of intervention effect by socio-economic position

The dichotomous SEP variable is entered into the analysis as the primary measure of SEP. Logistic regression analysis is conducted with proportion of participants taking snacks as the outcome and proximity as the predictor. An interaction terms is included for proximity and SEP to investigate whether SEP moderates the proximity effect.

#### Perceived effort and perceived salience

Independent t-tests are conducted to analyse whether mean ratings of perceived effort and salience differ by proximity condition; alternatively, Mann–Whitney U tests are conducted if assumptions of normality are not met. A logistic regression model is conducted including perceived effort and salience of the M&Ms, with proportion of participants taking snacks as the outcome and proximity as the predictor, to investigate whether these variables affect the primary outcome. Further analyses includes exploration of the potential mediation of perceived effort and perceived salience on any observed main effects of proximity intervention on the proportion of participants taking any snacks.

#### Covariates

Liking for chocolate has been found to affect intake of chocolate M&Ms [8] and is therefore included as a covariate in all analysis. Hunger level is included in all the above analysis since this may affect intake of the M&Ms. Age is included as a covariate in the analyses since age may relate to education level and EF.

#### Treatment of participants who move the bowl

Because participants are not restricted from moving the bowl, this presents a potential issue whereby participants move the bowl and are then influenced by subsequent positions of the bowl rather than the initial position determined by the researcher as per protocol. We intend firstly to examine the effect of proximity using an intention-to-treat approach, whereby participants are analysed according to the condition to which they are randomly assigned. However, should we find that participants do indeed move the bowl, we will conduct additional per-protocol analysis, including only participants who adhere to the protocol as intended i.e. where the position of the bowl as determined by the researcher remains unchanged throughout the experimental session.

## Discussion

The proposed study adds to the literature in two ways: 1. by investigating the effect of proximity on snack food intake in a larger general population sample consisting of both high and low SEP participants, appropriately powered to detect the predicted proximity effect, and 2. by estimating the extent to which the proximity effect is moderated by EF.

### Testing proximity in the general population

Previous studies investigating the proximity effect may have generated results that are not generalisable to general populations given investigators have tended to either purposefully recruit university staff and students [7–9, 12, 14, 15, 36] or have conducted studies in university or hospital cafeterias where the customers are likely to have been university staff, students or health professionals [10, 16] - see Table 1 in the Additional file 1 for sample population information in identified proximity studies. Above-average intelligence and continuing in education both relate to higher EF [47]. Therefore recruiting a sample population with a high proportion of university students and staff may lead to a disproportionate representation of high EF and high SEP, limiting understanding of the strength of the proximity effect in populations with lower SEP and EF who are in greater need of dietary change. The proposed study will recruit members of the general population, from both high and low SEP backgrounds.

Previous experimental studies investigating proximity are also often limited by small sample sizes, reducing the reliability of the results. Studies have tested as few as 12 or 17 participants in each condition [8, 14, 15], or 16 participants in total [9] – see Table 1 in the Additional file 1 for sample sizes in these studies. The aforementioned studies do not report sample size calculations to justify using these sample sizes. Based on an a priori power calculation, the current study will recruit 156 participants to allow us sufficient power to detect predicted effects in both primary and secondary outcomes.

#### Moderation of the proximity effect by executive function

The proposed study is, to our knowledge, the first to estimate the extent to which the proximity effect is moderated by EF. This study is powered on the primary outcome corresponding to Hypothesis 1 and is not powered to detect an interaction between EF and proximity in order to support or undermine Hypothesis 2.

According to a provisional power analysis based on limited available data, very large samples are required to detect such an interaction, which is unfeasible for this study. As such, whilst this study may detect an interaction, which is contrary to Hypothesis 2; if it does not detect an interaction, this does not necessarily confirm Hypothesis 2 (i.e. it is possible that there is an interaction, though the study is not adequately powered to detect it). However, this study should provide the best available data to inform powering of future studies, building a foundation towards a stronger and more solid evidence base to inform Hypothesis 2.

If results are consistent with Hypothesis 2, the proximity effect should be present regardless of individual differences in EF. A similar effect of proximity on snack intake in both high and low EF participants would be compatible with the explanation that the proximity effect operates outside of the influence of EF. This suggests that proximity would be an effective intervention to alter selection and consumption of food with the potential to reduce SEP patterned behaviours such as poor diet that contribute to health inequalities.

## Conclusion

This study provides the first attempt to estimate the effect size of the proximity effect on consumption across social groups i.e. those from low as well as high SEP groups, and in relation to a potential cognitive moderator of this effect, namely executive function. Manipulating proximity, like other components of choice architecture, should ultimately be tested for its effectiveness to bring about sustained changes in dietary behaviour [6]. Therefore, should efficacy in laboratory settings be established, research should be extended from the laboratory to field settings such as shops or restaurants and with interventions implemented over extended time periods.

## Abbreviations

EF, Executive function; RIM, Reflective-Impulsive Model; SEP, Socio-economic position; VAS, Visual analogue scale.

## Additional files

Additional file 1: Table showing studies identified as experimentally investigating the proximity effect. (DOC 22 kb) Additional file 2: Table showing order of all questions and tests completed by participants. (DOC 28 kb)
