# Supplementary material for: Impact of altering proximity on snack food intake in individuals with high and low executive function: study protocol
Source: BMC Public Health. 2016 Jun 13;16:504. doi: 10.1186/s12889-016-3184-9 (PMC4906733; doi:10.1186/s12889-016-3184-9)
Supplement: Additional file 1: — Table showing studies identified as experimentally investigating the proximity effect. (DOC 22 kb) [file 12889_2016_3184_MOESM1_ESM.doc]

**Appendix 1: Table showing studies identified as experimentally investigating the proximity effect**

| Table 1 – Studies identified as testing the proximity effect | | | | | |
| --- | --- | --- | --- | --- | --- |
| **Authors** | **Sample Population** | **Testing environment** | **Conditions** | **Sample size** | **Findings** |
| Kroese et al. (2015) | Customers of shops | Train station snack shops | 1. Control  2. Nudge (proximity)  3. Nudge + disclosure (proximity + a sign “we help you make heathier choices”) | **Total N=91**  By condition:  1. n=30  2. n=30  3. n=31 | Healthy foods purchased more often when proximal at till with nudge (p<.001) and nudge + disclosure (P<.05) |
| Maas et al. (2012) | Female university students | Laboratory conditions | Between-subjects  **Study 1**  1. M&Ms proximal (20cm)  2. M&Ms within-reach (70cm)  3. M&Ms distal (140cm)  **Study 2**  1. M&Ms proximal (20cm)  2. M&Ms within reach (70cm)  3. M&Ms distal (140cm) | **Study 1 total N=77**  By condition:  1. n=23  2. n=26  3. n=28  **Study 2 total N=54**  By condition:  1. n=19  2. n=17  3. n=18 | **Study 1**  Higher % took M&Ms when proximal (proximal=74%, within-reach=42%, distal=21%)  Less likely to take snacks in within-reach compared to proximal (P<.05)  More M&Ms eaten when proximal (P<.001)  **Study 2**  Higher % took M&Ms when proximal  (proximal=79%, within-reach=35%, distal=44%)  Less likely to take snacks in within-reach compared to proximal (P<.05)  More M&Ms eaten when proximal (P<.05) |
| Meiselman et al. (1994) | University students | Cafeteria | Within-subjects  **Study 1**  Week 1. Candy at main tills (baseline)  Week 2. Candy at separate till 20m away (effort)  **Study 2**  Week 1-2. Crisps at main till (baseline)  Week 3-5. Crisps at separate till 20m away (effort)  Week 6-8. Recovery (same as baseline) | **Study 1 total N=43**  **Study 2 total N=60**  All subjects took part in all conditions | **Study 1**  Effort condition = reduction in candy selection (P<.001)  **Study 2**  Effort condition = reduction in crisp selection (P<.001) |
| Meyers et al. (1980) | Hospital staff, students & visitors | Cafeteria | 1. High and low calorie desserts alternate (control)  2. High calorie desserts at front  3. Low calorie desserts at front | **Total N=4412**  *Sample sizes per condition not reported* | Placement of dessert = dessert selection (P<.001) decrease in selection of low calorie desserts when at back row. No effect when high calorie desserts at rear  All weight status groups equally responsive to external food cues |
| Musher-Eizenman et al. (2010) | Children age 3.4-11yrs | Cafeteria | Within-subjects  1. 4 crackers (30 calories)  2. 4 carrot slices (8 calories)  *Distance is a continuous variable with each child varying in distance from serving bowl* | **Total** **N=46** | Less food intake with increasing distance for both crackers and carrots (P=.<05) |
| Painter et al. (2002) | University staff | Office | Within-subjects  1. Chocolates on top of desk  2. Chocolates in desk drawer  3. Chocolates on shelf 2m away | **Total** **N=16**  All subjects took part in all conditions | More chocolates eaten when closer (P<.01) |
| Privitera & Creary (2013) | University students | Kitchen | Between-subjects  2 food types: apples & carrots  1. In open bowl within arms-reach (proximate-visible)  2. In closed bowl within arms-reach (proximate-not visible)  3. In open bowl 2m away (distal-visible)  4. In closed bowl 2m away (distal-not visible) | **Total N=96**  n=48 to each food type  By condition:  1. n=12  2. n=12  3. n=12  4. n=12 | More apples (p < .001) and carrots (p < .04) eaten when closer |
| Privitera & Zuraikat (2014) | University students | Kitchen | Between-subjects  1. Apples near (30cm) popcorn far (200cm)  2. Popcorn near (30cm) apples far (200cm)  3. Both near (30cm) | **Total N=56**  By condition:  1. n=17  2. n=18  3. n=21 | More apples (p < .001) and popcorn (p < .001) eaten when closer  No effect of food preference on proximity |
| Rozin et al. (2011) | Customers of university cafeteria, primarily medical staff | Cafeteria | **Study 1**  1. Middle row (10inch from front)  2. Edge rows (at front)  **Study 2**  1. Middle row (10inch from front)  2. At each edge far end (2 access points)  **Study 3**  1. Middle row (10inch from front)  2. Edge row A (facing entrance)  3. Edge row B (facing exit) | **M=157 purchases per day**  *Sample sizes per condition not reported* | **Study 1**  Lower intake from middle row (P<.001)  **Study 2**  No effect of accessibility on intake  **Study 3**  Lower intake from middle row (p <.01) |
| Sigurdsson et al. (2011) | Customers of supermarkets | X2 Supermarkets  (1x discount store  1x convenience store) | Group alternating treatment design  A. Bananas at fruit shelf (baseline)  B. Bananas fruit shelf + checkout  C. Bananas fruit + sweet shelves  D. Combination of all the above | **Total N=174**  *Sample sizes per condition not reported* | In discount store: fruit placement at both checkout + sweets shelf increased intake of bananas (tenuous – no p-value given)  Effect not replicated in convenience store |
| Thorndike et al. (2012) | Customers of hospital cafeteria | Cafeteria | Phase 1. Labelling (traffic light colour system for healthiness of food)  Phase 2. Choice architecture (healthy foods at eye level) | **N=977,793 items sold during baseline**    *Sample sizes per condition not reported* | Reduction of less healthy food intake and increase of healthy food intake with choice architecture in combination with labelling (P<.001) |
| Vohs & Heatherton (2000) | Female university students | Laboratory | Between-subjects  **Study 1**  Temptation (Proximity):  1. Sweets & snacks next to participant  2. Sweets & snacks across room  Availability:  1. help yourself  2. don’t touch  **Study 2**  Same proximity conditions as above:  **Study 3**  Same proximity conditions as above: | **Study 1 total N=100**  n=36 dieters  n=64 non-dieters  **Study 2 total** **N=31**  Dieters only    **Study 3** **total N=39**  Dieters only  *Sample sizes per condition not reported* | **Study 1**  Increased intake in dieters when snacks closer when told “help yourself” (P<.05)  “marginally significant” 3-way interaction between dieting status, proximity and availability (facilitation)  **Study 2**  Closer food leads to lower self-regulation (P<.05)  **Study 3**  Greater self-regulation during task leads to depletion of self-regulatory resources, as indicated by increased intake of food later in the procedure (P<.03) |
| Wansink et al. (2006) | Female university staff | Office | Within-subjects  1. Proximal & visible snack  2. Proximal & non-visible snack  3. Less proximal &visible  4. Less proximal &non-visible | **Total N=40**  By condition:  1. n=10  2. n=10  3. n=10  4. n=10 | More chocolates eaten when closer and visible (P<0.05) |
