# Supplementary material for: Impact of altering proximity on snack food intake in individuals with high and low executive function: study protocol
Source: BMC Public Health. 2016 Jun 13;16:504. doi: 10.1186/s12889-016-3184-9 (PMC4906733; doi:10.1186/s12889-016-3184-9)
Supplement: Additional file 2: — Table showing order of all questions and tests completed by participants. (DOC 28 kb) [file 12889_2016_3184_MOESM2_ESM.doc]

| **Appendix 2: Table showing order of all questions and tests completed by participants** | | |
| --- | --- | --- |
| Table 2 - Order of Questionnaire Items | | |
| **Stage of study** | **Measures** | **Items in order of presentation** |
| **Distributed by agency and completed online** | | |
| **Screening** | **Consent** | **1**. a. I have read, understood and accept the Information for Participants and have had the opportunity to ask questions  b. I confirm that I am 18 years or older  c. I understand that my participation is totally voluntary. I am free to withdraw at any time without having to give a reason  d. I understand that all personal information will remain confidential and that all efforts will be made to ensure I cannot be identified  e. I agree that the data gathered in this study may be stored anonymously and securely, and may be used for future research  f. I agree to take part in the study  OR - g. I do NOT agree to take part in this research study. (You will not proceed with the study)  **2**. *Please note that if you do not meet the eligibility criteria for this study, we will be unable to invite you to take part in the testing session and will not be able to pay your incentive.*  Please tick the box below if you are happy to proceed to take the screening questionnaire  ☐Yes ☐ No |
|  | **Age** | **3** Your age (in years)  |
|  | **Gender** | **4.**Your gender  ☐Male ☐Female ☐Rather not say |
|  | **Education level (SEP)** | **5. What is the highest education qualification you have achieved?**  ☐No qualifications  ☐Up to 4 GCSE's (Including 1-4 O Levels/CSE/GCSEs (any grades), Foundation Diploma, NVQ level 1, Foundation GNVQ or equivalents) (or foreign equivalent)  ☐5 or more GCSE's or 1 A-level (Including 5+ GCSEs (Grades A*-C),1 A Level/ 2-3 AS Levels, NVQ level 2, Intermediate GNVQ, City and Guilds Craft, BTEC First/General Diploma, RSA Diploma, Apprenticeship or equivalents) (or foreign equivalent)  ☐2 or more A-levels (Including 2+ A Levels, 4+ AS Levels, NVQ Level 3, Advanced GNVQ, City and Guilds Advanced Craft, ONC, OND, BTEC National, RSA Advanced Diploma or equivalents) (or foreign equivalent)  ☐Bachelor's degree (Including BA, BSc, NVQ Level 4-5, HNC, HND, RSA Higher Diploma, BTEC Higher level or equivalents) (or foreign equivalent)  ☐Post-Graduate degree or qualification (Including Higher Degrees e.g. MA, PhD, PGCE, Professional qualifications e.g. teaching, nursing, accountancy or equivalents) (or foreign equivalent) |
|  | **Postcode** | **6. What is your postcode?**  |
|  | **Food allergies/**  **intolerance** | **7**.Do you have any food allergies or intolerances?  ☐Yes ☐No  If Yes - please provide details here: ___________________ |
| **Completed during the study session** | | |
| **EF measures (pre-relax)** | **Stroop Task** | Completed using Inquisit 4 software |
|  | **ID** | **1.**Please enter the ID you have been provided:  |
|  | **WebEXEC** | ***2.****Please rate the extent to which you have problems in the following areas by picking the appropriate option from the drop-down menu below each item.*   1. Do you find it difficult to keep your attention on a particular task? 2. Do you find yourself having problems concentrating on a task? 3. Do you have difficulty carrying out more than one task at a time? 4. Do you tend to "lose" your train of thoughts? 5. Do you have difficulty seeing through something that you have started? 6. Do you find yourself acting on "impulse"?   *Response options:*  ☐No problems experienced ☐A few problems experienced ☐More than a few problems experienced  ☐A great many problems experienced  *For question* ***f.*** *response options were changed to the following:*  ☐Not at all ☐Not very much ☐Quite a lot ☐ Very much |
|  | **Delay discounting** | **3**.Which would you rather have?  ☐£45, received in three days  ☐£70, received in three months |
| **10 minute relaxation break** | | |
| **EF Measures (post-relax)** | **Stroop** | Completed using Inquisit 4 software |
|  | **ID** | **1.**Please enter the ID you have been provided:  |
|  | **WebEXEC** | ***2.****Please rate the extent to which you have problems in the following areas by picking the appropriate option from the drop-down menu below each item.*   1. Do you find it difficult to keep your attention on a particular task? 2. Do you find yourself having problems concentrating on a task? 3. Do you have difficulty carrying out more than one task at a time? 4. Do you tend to "lose" your train of thoughts? 5. Do you have difficulty seeing through something that you have started? 6. Do you find yourself acting on "impulse"?   *Response options:*  ☐No problems experienced ☐A few problems experienced ☐More than a few problems experienced  ☐A great many problems experienced  *For question* ***f.*** *response options were changed to the following:*  ☐Not at all ☐Not very much ☐Quite a lot ☐ Very much |
|  | **Delay discounting** | **3**. Which would you rather have?  ☐£45, received in three days  ☐£70, received in three months |
| **Main Questionnaire** |  |  |
|  |  |  |
|  | **Awareness of study** | **4.** What do you think the study was about?  **5.** What do you think the aim of the research is?  **6**. Did anything you were asked to do or anything that was in the room affect your actions or how you were thinking?  If so, how?__________________________________________________________________________ |
|  | **Hunger** | **7. At the moment, how hungry are you?**   \| **1** \| **2** \| **3** \| **4** \| **5** \| **6** \| **7** \| \| --- \| --- \| --- \| --- \| --- \| --- \| --- \| \| **(Not at all)** \|  \|  \|  \|  \|  \| **(Very)** \| |
|  | **Chocolate liking** | **8.** How pleasant would it be to experience a mouthful of chocolate now?  Not at all ________________________________________________________extremely |
|  | **Effort** | **9. Read the following statements and indicate to what extent you agree with each statement:**   \|  \| 1 (completely disagree) \| 2 \| 3 \| 4 \| 5 (completely agree) \| \| --- \| --- \| --- \| --- \| --- \| --- \| \| 1.It required effort to be able to get the M&Ms \|  \|  \|  \|  \|  \| \| 2.The M&Ms were directly within reach \|  \|  \|  \|  \|  \| \| 3. Before I could obtain the M&Ms, I first had to do something \|  \|  \|  \|  \|  \| \| 4.The M&Ms were effortlessly  obtainable \|  \|  \|  \|  \|  \| |
|  | **Salience** | **10. Read the following statements and indicate to what extent you agree with each statement:**   \|  \| 1 (completely disagree) \| 2 \| 3 \| 4 \| 5 (completely agree) \| \| --- \| --- \| --- \| --- \| --- \| --- \| \| 1.The M&Ms looked tempting \|  \|  \|  \|  \|  \| \| 2.I could hardly resist the M&Ms \|  \|  \|  \|  \|  \| \| 3.The M&Ms looked tasty \|  \|  \|  \|  \|  \| \| 4.The M&Ms looked irresistible \|  \|  \|  \|  \|  \| \| 5.I hardly noticed the M&Ms \|  \|  \|  \|  \|  \| |
|  | **Handedness** | **11. Please indicate your preferences in the use of hands in the following activities or objects:**   \|  \| Always Right \| Usually Right \| Both Equally \| Usually Left \| Always Left \| \| --- \| --- \| --- \| --- \| --- \| --- \| \| Writing \| ☐ \| ☐ \| ☐ \| ☐ \| ☐ \| \| Throwing \| ☐ \| ☐ \| ☐ \| ☐ \| ☐ \| \| Toothbrush \| ☐ \| ☐ \| ☐ \| ☐ \| ☐ \| \| Spoon \| ☐ \| ☐ \| ☐ \| ☐ \| ☐ \| \|  \|  \|  \|  \|  \|  \| |
|  | **Ethnicity** | **12. Your race/ethnicity**  ☐White British / White Irish / Other White background  ☐Mixed White and Black African / Mixed White and Asian / Mixed White and Black Caribbean / Other Mixed background  ☐Asian/Asian British (Indian/Pakistani/Bangladeshi/Chinese/Other Asian/Asian British background)  ☐Black/Black British (African/Caribbean/Other Black/Black British background)  ☐Other ethnic group |
|  | **Education** | **13. What is the highest education qualification you have achieved?**  ☐No qualifications  ☐Up to 4 GCSE's (Including 1-4 O Levels/CSE/GCSEs (any grades), Foundation Diploma, NVQ level 1, Foundation GNVQ or equivalents) (or foreign equivalent)  ☐5 or more GCSE's or 1 A-level (Including 5+ GCSEs (Grades A*-C),1 A Level/ 2-3 AS Levels, NVQ level 2, Intermediate GNVQ, City and Guilds Craft, BTEC First/General Diploma, RSA Diploma, Apprenticeship or equivalents) (or foreign equivalent)  ☐2 or more A-levels (Including 2+ A Levels, 4+ AS Levels, NVQ Level 3, Advanced GNVQ, City and Guilds Advanced Craft, ONC, OND, BTEC National, RSA Advanced Diploma or equivalents) (or foreign equivalent)  ☐Bachelor's degree (Including BA, BSc, NVQ Level 4-5, HNC, HND, RSA Higher Diploma, BTEC Higher level or equivalents) (or foreign equivalent)  ☐Post-Graduate degree or qualification (Including Higher Degrees e.g. MA, PhD, PGCE, Professional qualifications e.g. teaching, nursing, accountancy or equivalents) (or foreign equivalent) |
|  | **Postcode** | **14. What is your postcode?**  |
|  | **Height** | **15. Height:**  a. What units would you prefer to give your height in?  ☐cm  ☐feet and inches  b. Please provide your height:   \|  \|  \|  \|  \|  \| \| --- \| --- \| --- \| --- \| --- \| \|  \|  \|  \|  \|  \| |
|  | **Weight** | **16. Weight:**  a. What units would you prefer to give your weight in?  ☐kg  ☐stones and pounds  b. Please provide your weight:   \|  \|  \|  \|  \|  \| \| --- \| --- \| --- \| --- \| --- \| |
